# Typical carcinoid tumor of the larynx in a woman: a case report

**DOI:** 10.1186/1752-1947-4-321

**Published:** 2010-10-12

**Authors:** Fatma Tülin Kayhan, Efser Gürer Başaran

**Affiliations:** 1 Bakırköy Dr. Sadi Konuk Training and Research Hospital, Department of Otolaryngology, Istanbul, Turkey

## Abstract

**Introduction:**

Neuroendocrine tumors are the second most common neoplasms of the larynx. Histopathologically, neuroendocrine tumors can be classified into four types: typical carcinoid tumors, atypical carcinoid tumors, small cell neuroendocrine tumors and paragangliomas. Typical carcinoid tumor of the larynx is a particularly rare occurrence. We present a case of this rare disease, and review and discuss its diagnosis and treatment.

**Case presentation:**

A 55-year-old Turkish woman presented with a two-year history of persistent hoarseness. Endoscopic laryngeal examination and computed tomography revealed a supraglottic mass. Direct laryngoscopy was performed and a biopsy taken. Results of the histopathologic examination and immunohistochemical analysis were consistent with typical carcinoid tumor of the larynx. A supraglottic laryngectomy was performed. There was no recurrence during a follow-up period of three years.

**Conclusion:**

Carcinoid tumors require an accurate diagnosis because of their varied clinical behavior and prognosis. A correct pathologic diagnosis is essential, differentiating the tumors from other neuroendocrine neoplasms and medullary cancer of the thyroid gland. Immunohistochemical analysis is supplementary to a standard histopathologic evaluation. Currently, conservative surgical resection without elective neck dissection is the recommended treatment for typical carcinoid tumor of the larynx. Additional cases and case series with long-term follow-up will be useful for understanding the nature of this tumor and should clarify the prognosis.

## Introduction

Neuroendocrine tumors of the larynx are the second most common neoplasm of the larynx after squamous cell carcinomas. Four types of neuroendocrine tumor have been identified by the World Health Organization (WHO): typical carcinoid tumor, atypical carcinoid tumor, small-cell neuroendocrine carcinoma and paraganglioma [1-3]. Carcinoid tumors and small cell neuroendocrine tumors originate from epithelium, whereas paragangliomas originate from neural tissue. Typical carcinoid tumors are less common than other neuroendocrine tumors of the larynx, with approximately 20 cases reported in the English language literature to date [4]. We present an unusual case of typical carcinoid tumor of the larynx in a woman, and review the literature on neuroendocrine neoplasms of the larynx.

## Case report

A 55-year-old Turkish woman presented with a two-year history of hoarseness. She did not use tobacco or alcohol, and other than a three-year history of diabetes mellitus, her medical history was unremarkable. Her family history was non-contributory.

On endoscopic laryngeal examination, a supraglottic mass was seen, involving the laryngeal surface of the epiglottis and medial aspect of the pyriform sinus. Mobility of the vocal cords was normal. Examination of the neck found no palpable lymphadenopathy. No other abnormalities were found during the remainder of the otorhinolaryngological examination, or the general physical and pulmonary examinations. The results of laboratory tests were normal, including complete blood count, Westergren erythrocyte sedimentation rate, glucose level and electrolytes.

**Figure 1:**
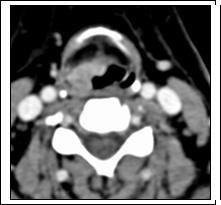
**Computed tomography with intravenous contrast showed a supraglottic mass**.

Cervical computed tomography (CT) showed a supraglottic mass extending from the right aspect of the medial glossoepiglottic fold to the right aryepiglottic fold and pyriform sinus, with involvement of the parapharyngeal fat tissue (Figure 1). No lymphadenopathy was seen on cervical CT. Thoracic and abdominal CT revealed no metastases or synchronous lesions.

**Figure 2:**
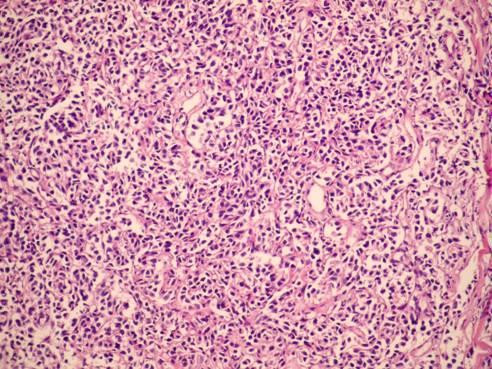
**The lamina propria contained a tumor consisting of small trabeculae and small nests of round cells (hematoxylin and eosin, original magnification ×100)**.

**Figure 3:**
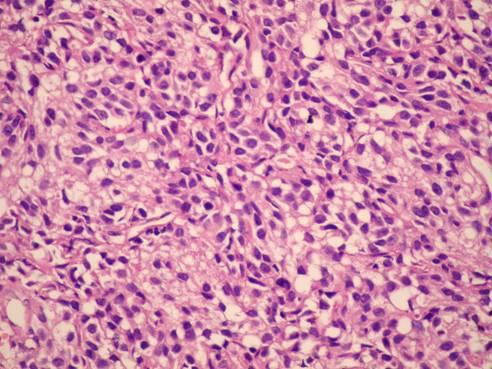
**Uniform round cells with salt and pepper chromatin without nucleoli, mitoses, or pleomorphism (hematoxylin and eosin, original magnification ×400)**.

**Figure 4:**
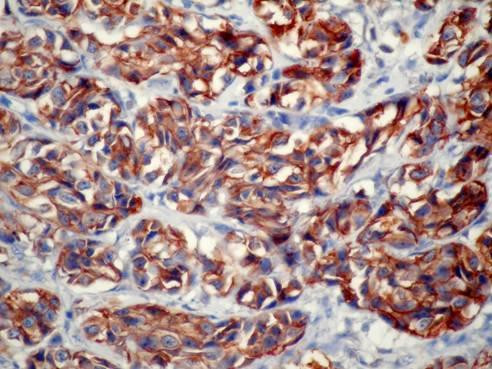
**Tumor cells were strongly immunopositive for chromogranin (anti-chromogranin, original magnification ×400)**.

Direct suspension laryngoscopy was performed under general anesthesia. A laryngeal mass with a smooth surface was seen. Multiple punch biopsies were taken from the mass, and histopathologic examination was performed. The tissues were stained with hematoxylin and eosin and evaluated by light microscopy. Pathological examination of the biopsy showed tumor infiltration, with trabeculae and small nests of round eosinophilic cells with uniform hyperchromatic nuclei, displaying a 'salt and pepper' chromatin pattern. There was no marked pleomorphism or necrosis (Figure 2 and Figure 3). The cells were strongly immunopositive for neuron-specific enolase, chromogranin (Figure 4), synaptophysin and pancytokeratin. Light microscopy and immunohistochemical analysis revealed typical carcinoid tumor of the larynx.

A supraglottic horizontal laryngectomy with a tracheotomy was performed. The histopathologic diagnosis was not changed postoperatively, and the surgical margins were tumor-free. There were no intraoperative or postoperative complications. The tracheotomy was closed in the second postoperative week. Postoperatively, the patient did not receive radiation therapy or chemotherapy. There was no recurrence during a follow-up period of three years.

## Discussion

Four different types of neuroendocrine tumor of the larynx have been identified by the WHO: typical carcinoid tumor, atypical carcinoid tumor, small-cell neuroendocrine tumor and paraganglioma [2,3,5].

A definite diagnosis can only be made histopathologically. An accurate histologic diagnosis is essential because the treatment and prognosis depend on the type of neuroendocrine tumor [2].

Typical carcinoid tumor in the larynx is very uncommon. This tumor type represents 0.5% to 1% of all epithelial neoplasms, and is the least common neuroendocrine tumor of the larynx. To our knowledge, the case presented here is only the 20^th ^reported in the English language literature [5,6].

There is a slight male preponderance for this tumor, with a male to female ratio of 3:1 [2,5]. Typically, patients with this tumor are in the sixth to eighth decade of life, and are smokers [2,5,6]. Unusually, our patient was a woman in her fifties, who did not have a history of smoking, dysphagia or odynophagia [2,5]. She had only hoarseness as a symptom, and endoscopic examination revealed a supraglottic mass with a smooth surface. A typical carcinoid tumor generally grows submucosally, and is seen as a polypoid or sessile mass [5]. Neuroendocrine tumors of the larynx are located in the supraglottis because the supraglottic area contains abundant neuroendocrine cells [5].

The diagnostic investigation should include indirect fiberoptic laryngoscopy, which will show a submucosal mass in the supraglottic larynx. CT of the larynx should be used to determine local and regional tumor spread [2].

The diagnosis is made histopathologically; it may be difficult to distinguish between typical and atypical carcinoid tumor using light microscopy. Typical carcinoid tumor is comprised of oval or round cells with uniform hyperchromatic nuclei, but without significant pleomorphism [2]. The cytoplasm is scant and contains eosinophilic inclusions [2]. Scattered, infrequent mitoses and hyaline stroma are seen [2]. Angiolymphatic invasion, pleomorphism and necrosis are not seen in typical carcinoid tumor [5-7].

Intense immunoreactivity for neuron-specific enolase, chromogranin A, synaptophysin and cytokeratin are the criteria for a histologic diagnosis [5]. The neuroendocrine neoplasms of the larynx contain neurosecretory components, and can cause paraneoplastic syndrome; however, this condition is rare [5].

The prognosis of typical carcinoid tumor is good. It should be treated using conservative surgery [2,5-7]. If the tumor is extensive and lymphatic metastasis has occurred, a total laryngectomy and elective neck dissection should be performed [2,5,7]. Typical carcinoid tumors are radioresistant [2,4]. Radiotherapy and chemotherapy are recommended for advanced cases. We performed only a supraglottic laryngectomy because the tumor was localized to the supraglottic area and there was no lymphatic metastasis.

Atypical carcinoid tumors of the larynx are more common and more aggressive than other neuroendocrine tumors of the larynx [1]. The clinical findings and location of atypical carcinoid tumors are similar to those of typical carcinoid tumors. A history of smoking is common. Histopathologically, pleomorphism, nuclear atypia, necrosis and vascular invasion are found in atypical carcinoid tumor. The prognosis of atypical carcinoid tumor is poorer than that of typical carcinoid tumor, with a 5-year survival rate of approximately 50% [1]. Extended surgical resection and elective neck dissection are proposed treatment options [1]. Radiotherapy and chemotherapy can be added to surgical treatment [1].

Small cell neuroendocrine neoplasm of the larynx has an extremely poor prognosis, with five-year survival rates of 5% [1]. Generally, cervical and distant metastases are seen at the time of diagnosis. Surgical treatment is not indicated, and chemotherapy and radiotherapy are the mainstays of treatment [1].

The prognosis of paraganglioma of the larynx is good [1]. This tumor should be treated with complete surgical excision; elective neck dissection is not necessary.

## Conclusions

Typical carcinoid tumor of the larynx is an extremely rare condition. Although the reported data suggest that a wide local excision is adequate treatment, the five-year survival rate is unclear. Additional cases and case series with long-term follow-up results will be useful for understanding the nature of this tumor and will clarify the prognosis.

## Consent

Written informed consent was obtained from the patient for publication of this case report and accompanying images. A copy of the written consent is available for review by the Editor-in-Chief of this journal.

## Competing interests

The authors declare that they have no competing interests.

## Authors' contributions

FTK performed the biopsy and surgery, FTK and EGB analyzed and interpreted the clinical data, and FTK was a major contributor in writing the manuscript. All authors read and approved the final version of the manuscript.
